# *Burkholderia ambifaria* XN08: A plant growth-promoting endophytic bacterium with biocontrol potential against sharp eyespot in wheat

**DOI:** 10.3389/fmicb.2022.906724

**Published:** 2022-07-28

**Authors:** Chao An, Saijian Ma, Chen Liu, Hao Ding, Wenjiao Xue

**Affiliations:** Research Center for Microbial Metabolites, Shaanxi Institute of Microbiology, Xi'an, China

**Keywords:** *Burkholderia ambifaria*, biological control, *Rhizoctonia cerealis*, colonization, antifungal activity

## Abstract

Plant growth-promoting bacteria (PGPB) have been considered promising biological agents to increase crop yields for years. However, the successful application of PGPB for biocontrol of sharp eyespot in wheat has been limited, partly by the lack of knowledge of the ecological/environmental factors affecting the colonization, prevalence, and activity of beneficial bacteria on the crop. In this study, an endophytic bacterium XN08 with antagonistic activity against *Rhizoctonia cerealis* (wheat sharp eyespot pathogenic fungus), isolated from healthy wheat plants, was identified as *Burkholderia ambifaria* according to the sequence analysis of 16S rRNA. The antibiotic synthesis gene amplification and ultra-performance liquid chromatography-quadrupole time-of-flight mass spectrometry (UPLC-QTOF-MS) analyses were used to characterize the secondary metabolites. The results showed that the known powerful antifungal compound named pyrrolnitrin was produced by the strain XN08. In addition, *B. ambifaria* XN08 also showed the capacity for phosphate solubilization, indole-3-acetic acid (IAA), protease, and siderophore production *in vitro*. In the pot experiments, a derivate strain carrying the green fluorescent protein (*GFP*) gene was used to observe its colonization in wheat plants. The results showed that GFP-tagged *B. ambifaria* could colonize wheat tissues effectively. This significant colonization was accompanied by an enhancement of wheat plants' growth and an induction of immune resistance for wheat seedlings, which was revealed by the higher activities of polyphenol oxidase (PPO), peroxidase (POD), and phenylalanine ammonia-lyase (PAL). As far as we know, this is the first report describing the colonization traits of *B. ambifaria* in wheat plants. In addition, our results indicated that *B. ambifaria* XN08 might serve as a new effective biocontrol agent against wheat sharp eyespot disease caused by *R. cerealis*.

## Introduction

The wheat sharp eyespot, caused predominately by the necrotrophic fungus *Rhizoctonia cerealis*, is one of the most destructive soil-borne fungal diseases in wheat (*Triticum aestivum* L.) and results in yield losses of 10%−40% in the regions of Asia, Oceania, Europe, North America, and Africa (Wang et al., 2018; Zhao et al., 2021). This fungal pathogen can survive in soils or the infected crop residues for a long time, and it reinfects the stems and sheaths of wheat plants in the favorable environmental conditions, blocks the transportation of nutrients, and eventually leads to host death (Su et al., 2020). Traditional agrochemicals, which were still widely used for the effective control of wheat sharp eyespot, had led to an increase in environmental pollution and induced pesticide resistance (Zhang et al., 2017). Therefore, the biological control of wheat sharp eyespot as a green and sustainable agricultural biotechnology has attracted lots of attention (Raymaekers et al., 2020; Xu et al., 2020).

Plant growth-promoting bacteria (PGPB) have been considered promising biological agents for years (Dimkić et al., 2022). They have shown multifunctional plant-promoting ways including the facilitation of nutrient uptake, nitrogen fixation for plant use, the production of plant hormones, direct antagonism against pathogens, and the induction of systemic resistance throughout the plant (Jing et al., 2019). Therefore, many researchers have focused on the exploration of new PGPB with varied beneficial effects in recent years. For example, *Pantoea dispersa*-AA7 and *Enterobacter asburiae*-BY4, which were isolated from sugarcane rhizosphere soils, showed the capacity for nitrogenase and ACC deaminase production (Jing et al., 2019). Saad et al. (2020) isolated 18 strains from the rhizosphere soils of red silk-cotton tree and Chinese banyan and found that *Bacillus thuringiensis* MN419208 exhibited the capacity for plant growth promotion by producing indole-3-acetic acid (IAA) and exopolysaccharides and exerting the capacity of nitrogen fixation, while *Bacillus sonorensis* MN419205, *Bacillus wiedmannii* MN419207, and *Bacillus subtilis* MN419218 showed the antagonistic properties against root rot in *fava beans*. In contrast, a rhizosphere isolated strain of *Pseudomonas* sp. 23S showed antagonistic activity against *Clavibacter michiganensis* subsp. *michiganensis in vitro* and reduced the severity of tomato bacterial canker by inducing systemic resistance (Takishita et al., 2018). However, it is inadequate to excavate the wheat association PGPB, especially in screening the biological control agents against sharp eyespot caused by *R. cerealis*.

On the contrary, although many PGPB have shown excellent antagonistic characteristics under laboratory and greenhouse conditions, the successful application of PGPB under field conditions has been limited by its poor colonization capacity (Rilling et al., 2019). In fact, the effective root colonization of PGPB is considered to be a critical factor in achieving successful plant–microbe interaction (Bo et al., 2022). Compared with the plant rhizobacteria, bacterial endophytes have more opportunities to be in contact with the plant cells, so they could readily exert a direct beneficial effect (Morales-Cedeno et al., 2021).

In our previous study, an endophytic bacterium XN08, which showed great antagonistic activities against varied phytopathogenic fungi including *R. cerealis*, was isolated from healthy wheat plants. The purpose of this study was to evaluate its biocontrol potential against sharp eyespot in wheat. The strain XN08 was identified *via* 16S rRNA analysis, and a known antifungal compound produced by the strain was confirmed by gene amplification and ultra-performance liquid chromatography-quadrupole time-of-flight mass spectrometry (UPLC-QTOF-MS) analyses. To observe its colonization in wheat plants, a derivate strain carrying the green fluorescent protein (*GFP*) gene was constructed. The immune resistance of wheat seedlings was also monitored in this study.

## Materials and methods

### Bacterial strains and growth conditions

The strain XN08 used in this study was obtained from healthy wheat plants in our laboratory. The strain was cultivated in a nutrient broth medium (NB) and maintained at −80°C in a 20% glycerol solution. GFP-tagged *Burkholderia ambifaria* was cultivated in an NB medium containing 100 μg/ml tetracycline (Tc) for the maintenance of plasmids. *Rhizoctonia cerealis*, which was kindly provided by the Center of Biological Pesticide Research, Northwest Agricultural and Forestry University, was maintained on potato dextrose agar (PDA) slants. *Candida albicans*, which was derived from the Center of Microbiological Detection, Shaanxi Institute of Microbiology, was used as indicator fungi to detect the antifungal activity. The pyrrolnitrin was purchased from ChengDu TongChuangYuan Pharmaceutical Co. Ltd. (Chengdu, China). The Xiaoyan 22 (*T. aestivum* L.) seeds were directly purchased from the market.

### Phylogenetic analysis

The bacterial genomic DNA was isolated and purified using the TaKaRa MiniBEST Bacteria Genomic DNA Extraction Kit (Dalian, China). Genomic DNA was then used as the template for PCR amplification of 16S rRNA gene fragments using the bacterial universal primers (27F-5′-AGAGTTGATCCTGGCTCAG-3′ and 1492R-5′-GGTTACCTTGTTACGACTT-3′). The final amplified reaction volume was 50 μl, containing 5.0 μl of 10 × Taq buffers, 4.0 μl of 200 mmol/L dNTPs, 2.0 μl of each primer at 10 μM, 0.5 μl of Ex Taq enzyme (TaKaRa, Dalian), 5.0 μl of genomic DNA, and 31.5 μl of sterilized distilled water. PCR amplification was performed using the Professional Standard 96 Gradient (Biometra, Jena, Germany) with the following cycling parameters: initial denaturation of DNA for 5 min at 95°C, then 30 cycles of denaturation of DNA for 1 min at 94°C, annealing for 1 min at 53°C, extension for 1.5 min at 72°C, and final incubation for 5 min at 72°C (Vasiee et al., 2018). The PCR products were subsequently purified and sequenced using BGI Biotechnology (Shenzhen, China). DNA sequence alignment was performed using BLAST (https://blast.ncbi.nlm.nih.gov/Blast.cgi). Finally, phylogenetic trees were constructed using the neighbor-joining (NJ) method implemented in MEGA 5.05 (Arizona State University, Tempe, United States).

### Detection of genes associated with antibiotic biosynthesis using the PCR method

The *Burkholderia* spp. have been reported to produce antimicrobial compounds such as siderophore (required *Cep* gene), pyrrolnitrin (required *Prn* gene), and phenazine acid (required *Pca* gene). We designed three sets of low-degeneracy primers for PCR amplification of these genes. These primers are shown in Supplementary material 1. According to the manufacturer's instructions, the amplification was performed in 50 μl reactions with Taq polymerase (TaKaRa Biotechnology, Dalian, China). The PCR products were detected using gel electrophoresis detection. Finally, the PCR products obtained in this study were sequenced by BGI Biotechnology (Shenzhen, China), and the phylogenetic tree was constructed using the neighbor-joining (NJ) method.

### Evaluation of antifungal activity *in vitro* and *ex vivo*

Antifungal activity of the *Burkholderia* sp. XN08 was evaluated using both the dual plate confrontation assay and the agar diffusion method. The inhibition ratio was calculated as follows (Cui et al., 2019):


(1) $$\begin{array}{l} Inhibition\;ratio\;\left(\%\right)=\\ \frac{\left(Diameter\;of\;control\;fungus-Diameter\;of\;treated\;fungus\right)}{\left(Diameter\;of\;control\;fungus\right)}. \end{array}$$

The strain XN08, which was pre-cultured on a nutrient agar medium (NA) plate for 24 h, was inoculated into a 500-ml conical flask containing 200 ml of sterile LB broth and then cultured at 230 rpm for 48 h at 37°C. After cultivation, the cells were removed by centrifugation at 8,000 rpm for 10 min at 4°C. The supernatant was added into preheated PDA medium at 55°C. *Rhizoctonia cerealis* was respectively inoculated on the pure PDA medium plate and PDA medium supplemented with the fermentation broth supernatant of the strain XN08.

Plant leaf tissue was used to determine the antifungal activity of fermentation broth supernatant *ex vivo* as previously described (Fu et al., 2019) with minor modifications. In brief, the top leaves of 14-day-old wheat seedlings with four- to five-leaf stages were used for evaluating the biocontrol efficacy of the fermentation broth supernatant of the strain XN08 against *R. cerealis*. The leaves were wounded at the equator (0.5 mm wide) and inoculated with 5 μl of conidial suspension (2 × 10^5^ spores/ml) of *R. cerealis*. Then, the fermentation broth supernatant of the strain XN08 was sprayed onto the leaf surface. To keep the plant growing, the petioles were wrapped in cotton that contained water to provide nutrients. The leaves were kept in greenhouse system at 23 ± 2°C with a relative humidity (RH) of 95% for 6 days. Daily observations were carried out.

### Extraction and identification of antifungal compounds by UPLC-QTOF-MS

The *n*-butanol extracts from the fermentation broth supernatant of XN08 were dissolved with methanol at a concentration of 1 mg/ml, and 50 μl of the solution was added into a 6-mm well in Sabouraud agar medium plates, which were inoculated with *C. albicans* and cultured overnight at 37°C. Then, the plates were incubated at 28°C for 72 h, and the inhibition zones around the wells were observed to determine the antimicrobial activity. The redissolved sample was further purified using reverse-phase high-performance liquid chromatography (Waters 2695, PDA detector 2998) with a C18 column (YMC-Pack Pro C18, 250 × 4.6 mm S-5 μm, 12 nm; YMC CO., LTD, Japan) and eluted with a methanol–water mixture (methanol:water = 8:2) at a flow rate of 1.0 ml/min. The OD at 254 nm was monitored. The MS was operated in negative ion mode and was set to total ion chromatogram mode with the following parameter settings: capillary voltage, 1.0 kV; low collision energy, 6V; source temperature, 100°C; desolvation temperature, 500°C; and desolvation gas flow, 800 L/h. Data acquisition and processing were conducted using Masslynx version 4.1 (Waters, Manchester, United Kingdom).

### Detection of plant growth-promoting traits *in vitro*

#### IAA detection

The strain XN08 was propagated overnight in 100 ml of LB medium and then supplemented with 1 ml of l-tryptophan solution with a concentration of 50 μg/ml. After incubation for 42 h, 1 ml aliquot of the supernatant was mixed vigorously with 4 ml of Salkowski's reagent (150 ml of concentrated H_2_SO_4_, 250 ml of distilled H_2_O, and 7.5 ml of 0.5 M FeCl_3_∙6H_2_O) and allowed to stand at room temperature for 20 min, and then the color changes were observed (Patten and Glick, 2002).

#### Phosphate solubilization detection

A single colony of strain XN08 was spot inoculated onto Pikovskaya's agar plate at 28°C and incubated at 28°C for 48 h. The formation of a halo-zone around the colony was observed (Patten and Glick, 2002).

#### Siderophore detection

A single colony of strain XN08 was spot inoculated on CAS medium [medium component (1L): chrome azurol S (CAS), 60.5 mg; hexadecyltrimetyl ammonium bromide (HDTMA), 72.9 mg; piperazine-1, 4-bis (2-ethanesulfonic acid; PIPES), 30.24 g; and 1 mM FeCl_3_∙6H_2_O in 10 mM HCl 10 ml agarose (0.9%, w/v)], and then color changes around the colonies were observed visually (Shahid et al., 2012).

#### Proteinase detection

A single colony of the strain XN08 was spot inoculated on a modified tryptic soy broth medium and then the zones of proteolysis around the colonies were observed visually (Shahid et al., 2012).

#### Pot experiments

Wheat seeds were surface-sterilized in 2.5% sodium hypochlorite for 5 min and in 75% ethanol for 2 min and then soaked in sterile-distilled water for 24 h. The sterilized wheat seeds were put into a sterile 10-ml glass bottle and then cultured for 7 days in the artificial climate box with a temperature of 28°C and 90% RH. The seedlings with consistent growth were selected for the pot experiments. All experiments have been conducted in a 10-ml glass bottle, and each experiment group consisted of four bottles, each of which contains three seedlings.

- **Group 1:** The seedlings were poured with a 2-ml suspension of GFP-tagged *B. ambifaria* (10^7^ CFU/ml).
- **Group 2:** The control group consisted of seedlings soaked in 2 ml of sterile water.
- **Group 3:** The seedlings soaked in a 2-ml of sterile water were scratched and inoculated with 500 μl of the suspension of *R. cerealis* containing 1 × 10^8^ CFU/ml spores.
- **Group 4:** The seedlings were poured with 2 ml suspension of GFP-tagged *B. ambifaria* (10^7^ CFU/mL) and inoculated with 500 μl of suspension of *R. cerealis* containing 1 × 10^8^ CFU/ml spores.

All of the treated seedlings were further cultured in an artificial climate box (RH 90% and temperature 28°C) and then tested for plant growth-promoting and biocontrol properties. Three seedlings were sampled for each treatment at different growth stages. The samples were washed and wiped dry, and then their fresh weight and shoot height were measured.

### Colonization of XN08 in wheat tissues

The seedlings of group 1 were scanned and imaged by CLSM (Laser Scanning Confocal Microscopy; DM6000, Leica Microsystems). The STED beam was generated by a 592-nm depletion beam. All images were detected using hybrid (HyD) detectors controlled by LAS-AX imaging software. All STED images were deconvolved using Huygens software (Scientific Volume Imaging) and analyzed offline using LAS AF Lite (Leica).

### The activities of defense enzymes in wheat

The activity levels of polyphenol oxidase (PPO), peroxidase (POD), and phenylalanine ammonia-lyase (PAL) were determined according to the instructions provided by the manufacturer with the respective enzyme activity assay kits (Nanjing Jiancheng Bioengineering Institute, China). The article numbers of the kits are A136-1-1 (PPO), A084-3-1 (POD), and A137-1-1 (PAL).

## Results

### Identification of the strain XN08

**Figure 1:**
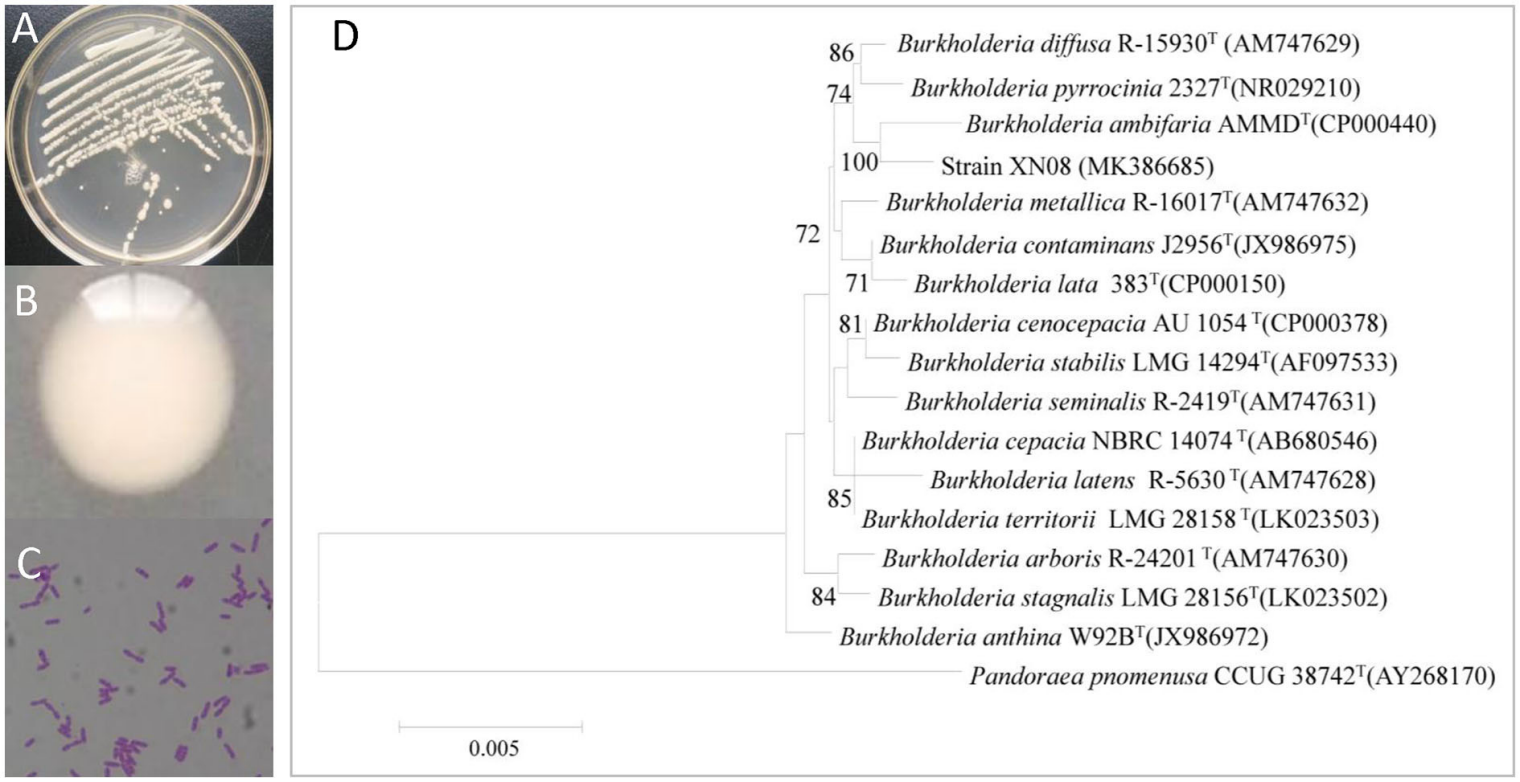
Morphological and molecular identification of the endophytic bacterium XN08. The colonies **(A)**, colony characters **(B)** of the strain XN08 on LB medium and microscopic characters **(C)** under a light microscope (×400), phylogenetic tree of the strain XN08 based on 16S rRNA sequence **(D)**.

As shown in Figures 1A,B, the strain XN08 exhibited off-white colony morphology and secreted viscoelastic substances on the NA plate. The cells appeared as short rod shapes under the light microscope (Figure 1C). A 1,438 bp region of the 16S rDNA gene was amplified from the genomic DNA of the strain XN08, and the sequence analysis indicated that the strain XN08 shared 99.85% identity with *B. ambifaria* AMMD (Genebank Accessions: CP00040) in the NCBI nr database. The phylogenetic tree was constructed (Figure 1D) using the neighbor-joining method. The result also showed that the strain XN08 had a close relationship with *B. ambifaria* AMMD. Therefore, the strain XN08 was identified as *B. ambifaria*.

### Biocontrol potential of the strain XN08 against *R. cerealis*

**Figure 2:**
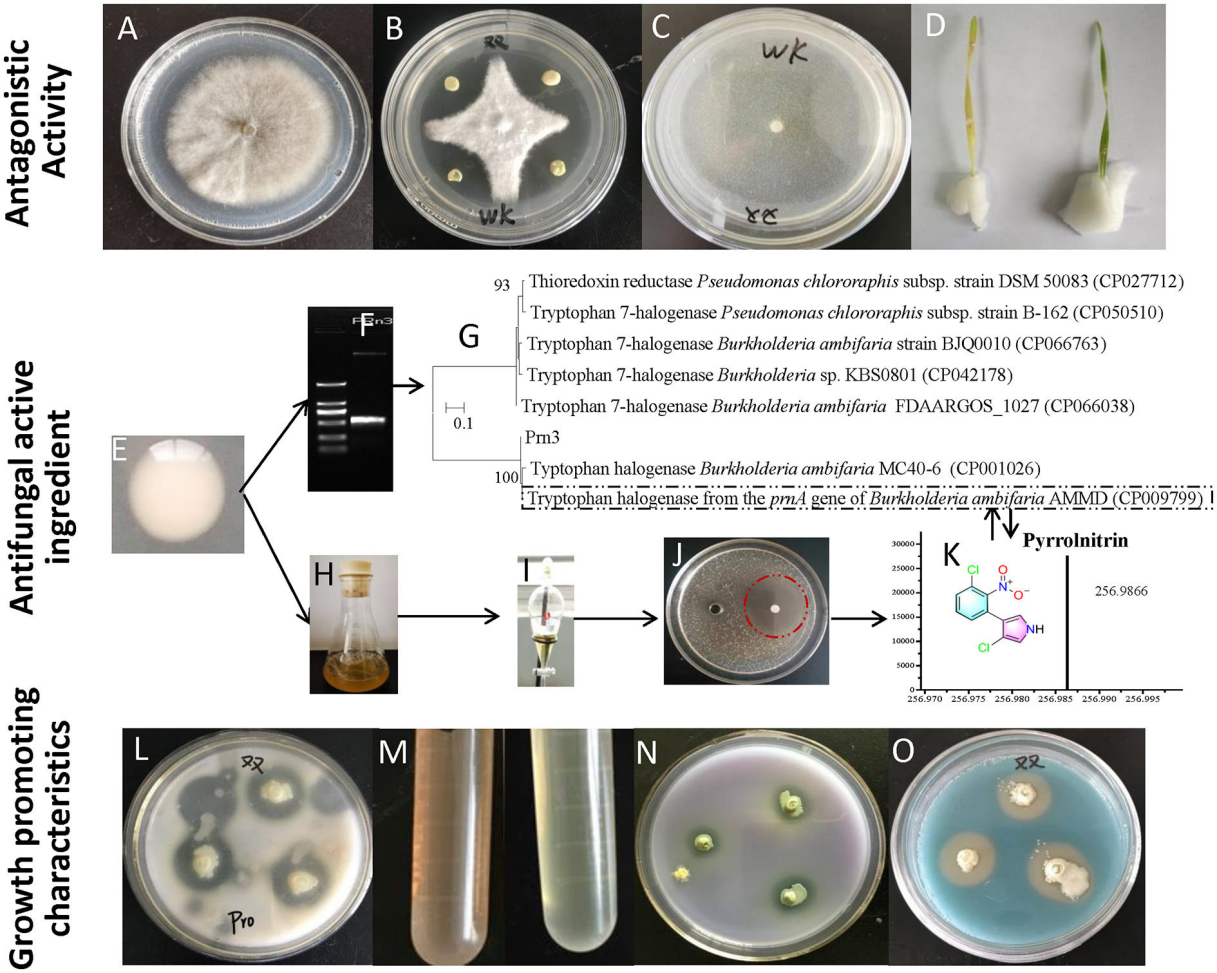
Antagonistic activities and plant growth-promoting characteristics of the strain XN08. *R. cerealis* grown on PDA medium **(A)**, *in vitro* antagonism of *B. ambifaria* XN08 against *R. cerealis* **(B)**, *R. cerealis* grown on PDA medium supplemented with the fermentation broth supernatant of *B. ambifaria* XN08 **(C)**, inhibition of the fermentation broth supernatant of the strain XN08 against *R. cerealis* on detached leaves of wheat plants **(D)**, amplification and sequence comparison of the antibiotic synthesis genes **(E–G)**, fermentation of the strain XN08 **(H)**, extraction of antifungal compounds **(I)**, detection of antifungal activities **(J)**, identification of antifungal compounds by UPLC-QTOF-MS method **(K)**, and plant growth-promoting traits of *B. ambifaria* XN08 *in vitro* [**(L)**, proteinase production; **(M)**, IAA production; **(N)**, the capacity of phosphate solubilization; **(O)**, siderophore production].

Compared with the control group (Figure 2A), *B. ambifaria* XN08 showed dramatically a high antagonistic activity against *R. cerealis* under the co-cultural condition (Figure 2B). In addition, no obvious *R. cerealis* growth was observed on the PDA medium added with the fermentation supernatants of the strain XN08 (Figure 2C). Moreover, the plant leaves sprayed with the supernatant of strain XN08 maintained a healthy green color, and no obvious disease symptoms were observed after inoculation with *R. cerealis* (Figure 2D). Usually, the leaves would become yellow after pathogen infection. The above results demonstrated that the strain XN08 may produce extracellular antifungal compounds.

### Identification of potential antifungal compounds

To predict the potential antifungal substances produced by *B. ambifaria* XN08, PCR was used to detect the biosynthetic genes of antifungal compounds. Three genes, namely, *Cep* R, *Prn*, and *Pca*, were amplified with predesigned three primers pairs, respectively. A key gene fragment sequence (*Prn* 3, 503 bp) involved in pyrrolnitrin synthesis was successfully amplified (Figures 2E,F). A phylogenetic tree was constructed by sequence alignment using the neighbor-joining method. The sequence had a close relationship with tryptophan halogenase from the *prn* A gene of *B. ambifaria* AMMD (CP009799; Figure 2G). The result indicated that *B. ambifaria* XN08 had the potential to synthesize pyrrolnitrin. An antifungal activity assay was performed to identify the fraction containing the antifungal compound. The *n*-butanol-extracted fraction of *B. ambifaria* XN08 fermentation broth (Figures 2H,I) exhibited obvious antifungal activity against *C. albicans* (Figure 2J). Subsequently, pyrrolnitrin was detected in the *n*-butanol-extracted fraction using the UPLC-QTOF-MS method (Figure 2K).

### *In vitro* plant growth-promoting traits of the strain XN08

As shown in Figures 2L–O, the strain XN08 exhibited a series of potential plant growth-promoting traits including protease, IAA, and siderophore production. Also, the strain could solubilize phosphate.

### Evaluation of plant colonization of GFP-tagged *B. ambifaria*

**Figure 3:**
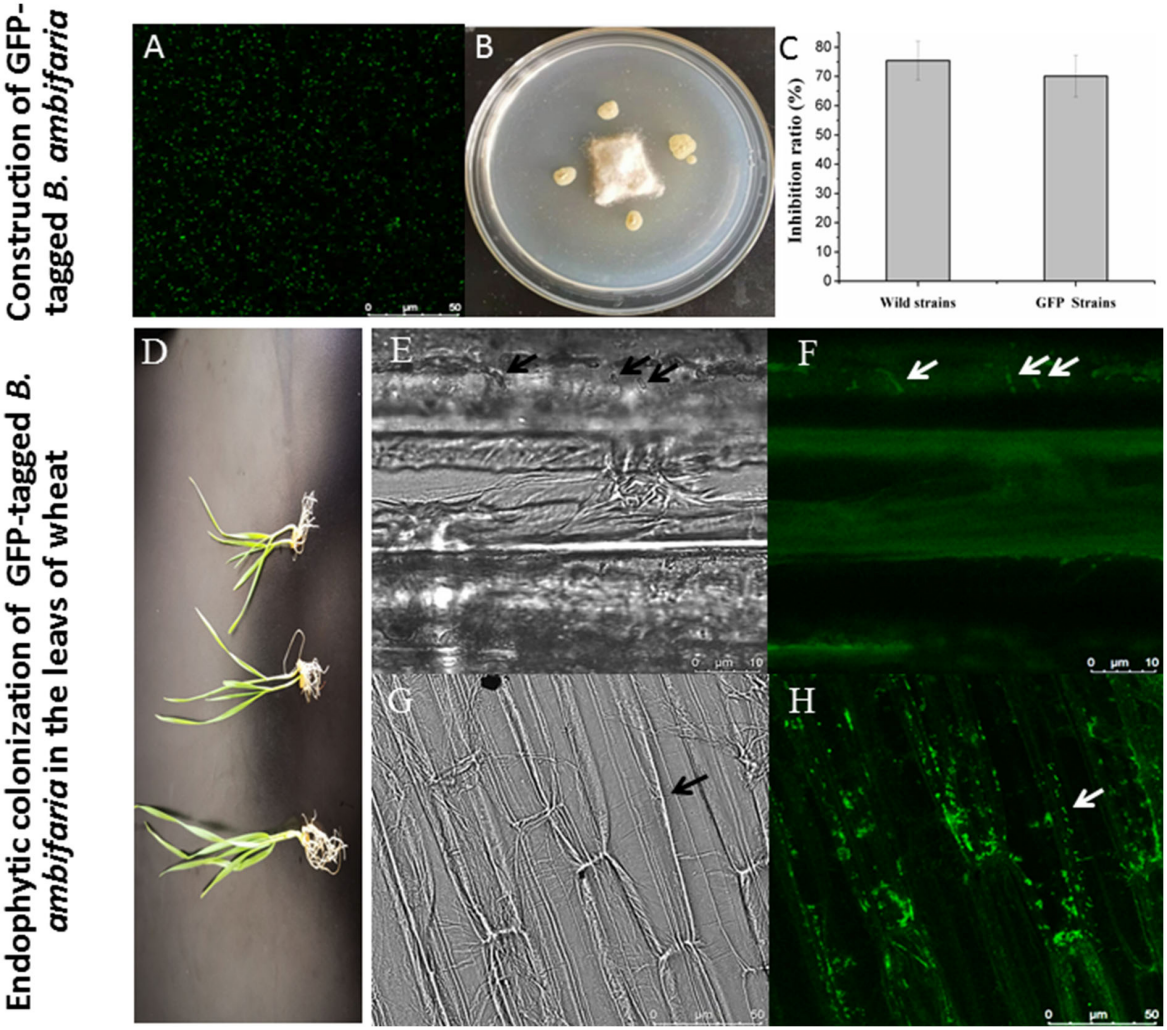
Colonization of GFP-tagged *B. ambifaria* in the seedlings of wheat. The observation of GFP-tagged *B. ambifaria* by CLSM **(A)**, antagonistic activities against *R. cerealis* **(B,C)**, the wheat seedlings inoculated GFP-tagged *B. ambifaria* at different growing stages **(D)**, colonization *of* GFP-tagged *B. ambifaria* XN08 at 2 days after inoculation (photographs under dark **(E)** and bright **(F)** fields) and 7 days after inoculation (photographs under dark **(G)** and bright **(H)** fields).

A derivative strain carrying the *GFP* gene was successfully constructed, and CLSM was used to observe the cells with green fluorescence as shown in Figure 3A. The GFP-tagged *B. ambifaria* showed a slightly weaker antagonistic activity (inhibition ratio of 70.14%) against *R. cerealis* compared with the wild strain XN08 (inhibition ratio of 75.45%; Figures 3B,C). In the pot experiment (Figure 3D), a small number of GFP-tagged *B. ambifaria* cells were found to colonize the root of wheat when the strain was inoculated into the rhizosphere soil of plants for 2 days (Figures 3E,F). At 7 days after inoculation, large numbers of bacterial cells were observed in the root tips (Figures 3G,H).

### Growth-promoting effects of *B. ambifaria* XN08 on wheat in the pot experiments

**Figure 4:**
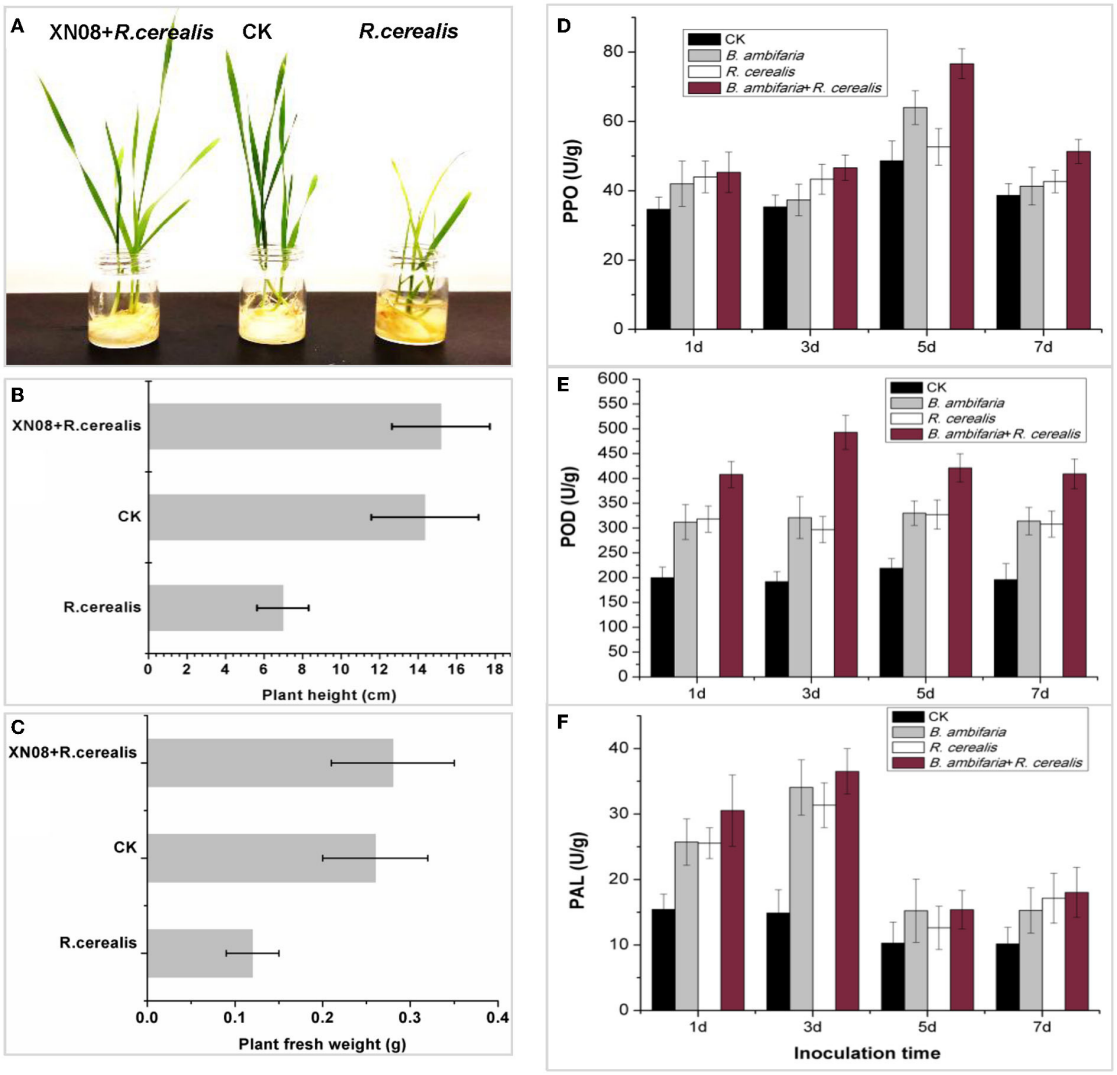
Antagonistic activities and plant growth-promoting characteristics of the strain XN08 in the pot experiments. Morphological changes in wheat seedlings **(A)**, plant heights **(B)**, plant fresh weights **(C)**, and the activities of defense enzymes, including PPO **(D)**, POD **(E)**, and PAL **(F)** under different treatments.

After 7 days of inoculation, the biological characteristics of the plant changed significantly as shown in Figure 4A. First, shoot height and fresh weight of the seedlings inoculated with both *R. cerealis* and GFP-tagged *B. ambifaria* reached 15.18 ± 2.54 cm and 0.28 ± 0.07 g for each seedling, which were higher than those of the seedlings inoculated with equal volumes of water (14.35 ± 2.78 cm, 0.26 ± 0.06 g) and the seedlings inoculated with *R. cerealis* (6.98 ± 1.34 cm and 0.12 ± 0.03 g) (Figures 4B,C). No significant difference was observed between the wheat seedlings with or without *R. cerealis* when inoculated with GFP-tagged *B. ambifaria*. In addition, the seedlings inoculated with *R. cerealis* had obvious sharp eyespot symptoms, while those plants inoculated with both *R. cerealis* and GFP-tagged *B. ambifaria* had no obvious symptoms.

The PPO, POD, and PAL enzyme activities of wheat seedlings at different growth stages for different treatment groups were tested. As shown in Figures 4D–F, the activities of PPO, POD, and PAL in the roots of wheat inoculated with both GFP-tagged *B. ambifaria* and *R. cerealis* were higher than those of the other groups and showed peaks at 5, 3, and 3 days after inoculation, respectively.

## Discussion

Wheat hosts a high diversity of endophytic bacteria, including different genera such as *Achromobacter, Acinetobacter, Arthrobacter, Bacillus, Burkholderia, Chitinophaga, Enterobacter, Erwinia, Flavobacterium, Klebsiella, Leifsonia, Microbispora, Micrococcus, Micromonospora, Mycobacterium, Paenibacillus, Pantoea, Pseudomonas, Roseomonas, Staphylococcus, Streptomyces*, and *Xanthomonas* (Rana et al., 2020). Several studies have exploited wheat-associated PGPB. It was reported that 13 endophytic bacteria isolated from wheat showed multifarious plant beneficial traits with the capacity of P-solubilization, nitrogenase, and IAA production (Rana et al., 2020). Larran et al. (2016) isolated the endophytes from wheat cultivars and found that the endophytes of *Penicillium* sp., *Bacillus* sp., and *Paecilomyces lilacinus* significantly suppressed the growth of pathogens *in vitro* and have the potential to be developed as new biological agents against the tan spot of wheat. In this study, the endophytic bacterium *B. ambifaria* XN08, which was isolated from healthy wheat plants, showed potential as a novel biological control agent against the wheat sharp eyespot. After all, only *Bacillus* spp. were exploited as biocontrol agents against *R. cerealis* in the previous reports (Ji et al., 2019; Yi et al., 2022).

*Burkholderia* is a genus of gram-negative bacteria with a wide environmental and geographic distribution (Estrada-De los Santos et al., 2001). Over the last years, there was an increasing interest in the genus *Burkholderia* due to its great potential value in plant growth promotion, biocontrol of plant pathogens, and phytoremediation (Bach et al., 2021). It was reported that bacterial endophytes belonging to *Bacillus* and *Burkholderia* genera were the most effective isolates in controlling bacterial and fungal pathogens *in vitro* (Morales-Cedeno et al., 2021). In fact, *Burkholderia* genus has rich antibiotic synthesis genes (Kim et al., 2021), and it has been reported to produce a large number of antifungal substances such as pyrrolnitrin, siderophores, and phenazines (Mullins et al., 2019), all of which play important roles in controlling fungal diseases in plants. In this study, a known powerful antifungal compound named pyrrolnitrin, production by XN08, was found, proving that the strain is valuable in controlling diseases including the wheat sharp eyespot. In addition, it is quite interesting that pyrrolnitrin production is quorum-sensing regulated, which indicates that efficient environmental colonization is crucial for the effective control of the wheat sharp eyespot disease (Chapalain et al., 2013).

Plant growth promotion and environmental colonization characteristics also determine the great potential of the *Burkholderia* genus as biocontrol agents (Paungfoo-Lonhienne et al., 2016). To observe the bacterial colonization directly and conveniently, a derivate strain carrying *GFP* gene was constructed in this study. The GFP-tagged *B. ambifaria* showed a similar inhibition ratio against *R. cerealis* as the wild-type strain, which indicated that the derivate strain could be used to efficiently observe its colonization in wheat plant tissue. Fluorescent labeling technology was the most intuitive way of representing the colonization of strain and was widely used in studying the plants and microorganisms interaction (Rilling et al., 2019; Sa et al., 2021). A number of potential biocontrol strains, including *Pseudomonas fluorescens, Paenibacillus glycanilyticus, Burkholderia tropica*, and *Bacillus velezensis*, labeled with a green fluorescent protein have been used to study their colonization capacity in plant tissue (Bernabeu et al., 2015; Kang et al., 2018; Li et al., 2019; Elsayed et al., 2020). However, as far as we know, this is the first time that *B. ambifaria* was labeled with green fluorescent protein to study the colonization characterized in this study.

It was reported that PGPB induced plants defense-related genes expression such as phenylalanine ammonia-lyase (PAL), catalase (CAT), polyphenol oxidase (PPO), peroxidase (POD), and superoxide dismutase (SOD), which might assist the plant to protect from or reduce the impact of pathogen attacks (Jiang et al., 2019; Kamou et al., 2019; Wu et al., 2019; Zhu et al., 2019; Ashajyothi et al., 2020; Singh et al., 2021). Our results showed that the significant colonization of XN08, visually observed with CLSM, was accompanied by the higher activities of PPO, POD, and PAL, which indicated that *B. ambifaria* XN08 was involved in the immune-induced resistance of wheat seedlings. In addition, the results also showed that the endophytic bacterial strain XN08 could significantly improve the growth of wheat inoculated with *R. cerealis*. The fresh weight and shoot height of the seedlings inoculated with both *R. cerealis* and GFP-tagged *B. ambifaria* were similar to those of the control group. Meanwhile, no obvious sharp eyespot symptom was observed for the group seedlings inoculated with both *R. cerealis* and GFP-tagged *B. ambifaria*. It is obvious that XN08 may provide an adequate protection against wheat disease.

On a whole, the results obtained in this study revealed that XN08 had great potential for biological control against the wheat sharp eyespot. However, it is worth noting that the successful application of XN08 for biocontrol of sharp eyespot in wheat needs knowledge of the ecological/environmental factors affecting the colonization of crops. Therefore, further observation using GFP-tagged *B. ambifaria* under different environmental conditions should be performed.

## Conclusion

Taken together, it was concluded that *B. ambifaria* XN08 was able to efficiently inhibit the growth of *R. cerealis* by producing antifungal compounds and showed the capacity to enhance plant growth by synthesizing a series of plant growth regulators. In addition, this strain exhibited significant colonization in wheat plants, which was accompanied by an enhancement of wheat plants' growth and an induction of immune resistance for wheat seedlings. These data indicated that the strain XN08 might be used as a new biocontrol agent against wheat sharp eyespot.

## Data availability statement

The original contributions presented in the study are included in the article/Supplementary materials, further inquiries can be directed to the corresponding author.

## Author contributions

CA performed the experiments, wrote, and edited the manuscript. WX designed and supervised the project. All experiments were also performed by SM, CL, and HD. All authors contributed to the article and approved the submitted version.

## Funding

This study was simultaneously supported by the National Natural Science Foundation of China (21576160) and the Scientific and Technologic Research Program of Shaanxi Academy of Sciences, China (No. 2018K-09).

## Conflict of interest

The authors declare that the research was conducted in the absence of any commercial or financial relationships that could be construed as a potential conflict of interest.

## Publisher's note

All claims expressed in this article are solely those of the authors and do not necessarily represent those of their affiliated organizations, or those of the publisher, the editors and the reviewers. Any product that may be evaluated in this article, or claim that may be made by its manufacturer, is not guaranteed or endorsed by the publisher.

## References

[B1] Ashajyothi M. Kumar A. Sheoran N. Ganesan P. Gogoi R. Subbaiyan G. K.. (2020). Black Pepper (*Piper Nigrum* L.) associated endophytic *Pseudomonas putida* BP25 alters root phenotype and induces defense in rice (*Oryza Sativa* L.) against blast disease incited by *Magnaporthe oryzae*. Biol. Control. 143, 104181. 10.1016/j.biocontrol.2019.104181

[B2] Bach E. Passaglia L. M. P. Jiao J. J. Gross H. (2021). *Burkholderia* in the genomic era: from taxonomy to the discovery of new antimicrobial secondary metabolites. Crit. Rev. Microbiol. 48, 121–160. 10.1080/1040841X.2021.1946009 34346791

[B3] Bernabeu P. R. Pistorio M. Torres-Tejerizo G. Estrada-De los Santos P. Galar M. L. Boiardi J. L.. (2015). Colonization and plant growth-promotion of tomato by *Burkholderia tropica*. Sci. Hortic-amsterdam. 191, 113–120. 10.1016/j.scienta.2015.05.01435575471

[B4] Bo T. T. Kong C. X. Zou S. X. Mo M. H. Liu Y. J. (2022). *Bacillus nematocida* B16 enhanced the rhizosphere colonization of *Pochonia chlamydosporia* ZK7 and controlled the efficacy of the root-knot nematode *Meloidogyne incognita*. Microorganisms. 10, 218. 10.3390/microorganisms10020218 35208675 PMC8879550

[B5] Chapalain A. Vial L. Laprade N. atacha. Dekimpe V. Perreault J. Déziel E. (2013). Identification of quorum sensing-controlled genes in *Burkholderia ambifaria*. MicrobiologyOpen 2, 226–242. 10.1002/mbo3.67 23382083 PMC3633348

[B6] Cui W. Y. He P. J. Munir S. He P. B. Li X. Y. Li Y. M.. (2019). Efficacy of plant growth promoting bacteria *Bacillus amyloliquefaciens* B9601-Y2 for biocontrol of southern corn leaf blight. Biol. Control. 139, 104080. 10.1016/j.biocontrol.2019.104080

[B7] Dimkić I. Janakiev T. Petrović M. Degrassi G. Fira D. (2022). Plant-associated *Bacillu*s and *Pseudomonas* antimicrobial activities in plant disease suppression *via* biological control mechanisms-a review. Physiol. Mol. Plant. P. 117, 101754. 10.1016/j.pmpp.2021.101754

[B8] Elsayed T. R Jacquiod S. Nour E. H. Sørensen S. J. Smalla K. (2020). Biocontrol of bacterial wilt disease through complex interaction between tomato plant, antagonists, the indigenous rhizosphere microbiota, and *Ralstonia solanacearum*. Front. Microbiol. 10, 2835. 10.3389/fmicb.2019.02835 31998244 PMC6967407

[B9] Estrada-De los Santos P. Bustillos-Cristales R. Caballero-Mellado J. (2001). *Burkholderia*, a genus rich in plant-associated nitrogen fixers with wide environmental and geographic distribution. Appl. Environ. Microbiol. 67, 2790–2798. 10.1128/AEM.67.6.2790-2798.2001 11375196 PMC92940

[B10] Fu M. R. Zhang X. M. Jin T. Li B. Q. Zhang Z. Q. Tian S.. (2019). Inhibitory of grey mold on green pepper and winter jujube by chlorine dioxide (ClO_2_) fumigation and its mechanisms. LWT 100, 335–340. 10.1016/j.lwt.2018.10.092

[B11] Ji P. Li W. G. Zheng Y. X. Wang Z. H. Huo Q. X. Hua C. Y. Han C. (2019). Isolation and identification of four novel biocontrol *Bacillus* strains against wheat sharp eyespot and their growth-promoting effect on wheat seedling. Int. J. Agric. Biol. 21, 282–288. 10.17957/IJAB/15.0892

[B12] Jiang C. H. Yao X. F. Mi D. D. Li Z. J. Yang B. Y. Zheng Y.. (2019). Comparative transcriptome analysis reveals the biocontrol mechanism of *Bacillus velezensis* F21 against *Fusarium Wilt* on Watermelon. Front. Microbiol. 10, 652. 10.3389/fmicb.2019.00652 31001229 PMC6456681

[B13] Jing L. M. Jeong J. C. Lee J. S Park J. M. Yang J. W. Lee M. H.. (2019). Potential of *Pantoea dispersa* as an effective biocontrol agent for black rot in sweet potato. Sci. Rep. 9, 16354. 10.1038/s41598-019-52804-3 31704990 PMC6841936

[B14] Kamou N. N. Cazorla F. Kandylas G. Lagopodi A. L. (2019). Induction of defense-related genes in tomato plants after treatments with the biocontrol agents *Pseudomonas chlororaphis* Toza7 and *Clonostachys rosea* Ik726. Arch. Microbiol. 202, 257–267. 10.1007/s00203-019-01739-4 31605156

[B15] Kang X. X. Zhang W. L. Cai X. C. Zhu T. Xue Y. R. Liu C. H. (2018). *Bacillus velezensis* CC09: a potential 'vaccine' for controlling wheat diseases. Mol. Plant. Microbe. Interact. 31, 623–632. 10.1094/MPMI-09-17-0227-R 29372814

[B16] Kim S. Jo S. Kim M. S. Shin D. H. (2021). A study of inhibitors of D-*glycero*-β-d-*manno*-heptose-1-phosphate adenylyltransferase from *Burkholderia pseudomallei* as a potential antibiotic target. J. Enzyme. Inhib. Med. Chem. 36, 776–784. 10.1080/14756366.2021.1900166 33733972 PMC7993394

[B17] Larran S. Simón M. R. Moreno M. V. Santamarina Siurana M. P. Perell,ó A. (2016). Endophytes from wheat as biocontrol agents against tan spot disease. Bio. Control. 92, 17–23. 10.1016/j.biocontrol.2015.09.002

[B18] Li L. M. Zhang Z. Pan S. Y. Li L. Li X. Y. (2019). Characterization and metabolism effect of seed endophytic bacteria associated with peanut grown in South China. Front. Microbiol. 10, 2659. 10.3389/fmicb.2019.02659 31798570 PMC6865467

[B19] Morales-Cedeno L. R. Orozco-Mosqueda M. D. Loeza-Lara P. D. Parra-Cota F. I. de los Santos-Villalobos S. Santoyo G. (2021). Plant growth-promoting bacterial endophytes as biocontrol agents of pre- and post-harvest diseases: fundamentals, methods of application and future perspectives. Microbiol. Res. 242, 126612. 10.1016/j.micres.2020.126612 33059112

[B20] Mullins A. J. Murray J. A. H. Bull M. J. Jenner M. Jones C. Webster G.. (2019). Genome mining identifies cepacin as a plant-protective metabolite of the biopesticidal bacterium *Burkholderia ambifaria*. Nat. Microbiol. 4, 996–1005. 10.1038/s41564-019-0383-z 30833726 PMC6544543

[B21] Patten C. L. Glick B. R. (2002). Role of *Pseudomonas putida* indoleacetic acid in development of the host plant root system. Appl. Environ. Microb. 68, 3795–3801. 10.1128/AEM.68.8.3795-3801.2002 12147474 PMC124051

[B22] Paungfoo-Lonhienne C. Lonhienne T. G. A. Yeoh Y. K. Donose B. C. Webb R. I. Parsons J.. (2016). Crosstalk between sugarcane and a plant growth promoting *Burkholderia* species. Sci. Rep. 6, 37389. 10.1038/srep37389 27869215 PMC5116747

[B23] Rana K. L. Kour D. Kaur T. Sheikh T. Yadav A. M. Kumar V.. (2020). Endophytic microbes from diverse wheat genotypes and their potential biotechnological applications in plant growth promotion and nutrient uptake. *Proc. Natl. Acad. Sci*. India 90, 969–979. 10.1007/s40011-020-01168-0

[B24] Raymaekers K. Ponet L. Holtappels D. Berckmans B. Cammue B. P. A. (2020). Screening for novel biocontrol agents applicable in plant disease management – a review. Biol. Control. 144, 104240. 10.1016/j.biocontrol.2020.104240

[B25] Rilling J. I. Acuña J. J. Nannipieri P. Cassan F. Maruyama F. Jorquera M. A. (2019). Current opinion and perspectives on the methods for tracking and monitoring plant growth–promoting bacteria. Soil. Biol. Biochem. 130, 205–219. 10.1016/j.soilbio.2018.12.012

[B26] Sa R. B. Zhang J. L. Sun J. Z. Gao Y. X. (2021). Colonization characteristics of poplar fungal disease biocontrol bacteria n6-34 and the inhibitory effect on pathogenic fungi by real-time fluorescence quantitative PCR detection. Curr. Microbiol. 78, 2916–2925. 10.1007/s00284-021-02529-2 34047833

[B27] Saad M. M. G. Kandil M. Mohammed Y. M. M. (2020). Isolation and identification of plant growth-promoting bacteria highly effective in suppressing root rot in fava beans. Curr. Microbiol. 77, 2155–2165. 10.1007/s00284-020-02015-1 32372106

[B28] Shahid M. Hameed S. Imran A. Ali S. Elsas J. D. V. (2012). Root colonization and growth promotion of sunflower (*Helianthus Annuus* L.) by phosphate solubilizing *Enterobacter* sp. Fs-11. World. J. Microb. Biot. 28, 2749-2758. 10.1007/s11274-012-1086-2 22806201

[B29] Singh P. Singh R. K. Li H. B. Guo D. J. Sharma A. Lakshmanan P.. (2021). Diazotrophic bacteria *Pantoea dispersa* and *Enterobacter asburiae* promote sugarcane growth by inducing nitrogen uptake and defense-related gene expression. Front. Microbiol. 11, 600417. 10.3389/fmicb.2020.600417 33510724 PMC7835727

[B30] Su Q. Wang K. Zhang Z. Y. (2020). Ecotopic expression of the antimicrobial peptide dmamp1w improves resistance of transgenic wheat to two diseases: sharp eyespot and common root rot. Int. J. Mol. Sci. 21, 647. 10.3390/ijms21020647 31963767 PMC7014311

[B31] Takishita Y. Charron J. B. Smith D. L. (2018). Biocontrol rhizobacterium *Pseudomonas sp*. 23S induces systemic resistance in tomato (*Solanum lycopersicum* L.) against bacterial canker *Clavibacter michiganensis* subsp *michiganensis*. Front. Microbiol. 9, 2119. 10.3389/fmicb.2018.02119 30254615 PMC6141633

[B32] Vasiee A. Behbahani B. A. Yazdi F. T. Mortazavi S. A. (2018). Diversity and probiotic potential of lactic acid bacteria isolated from Horreh, a traditional Iranian fermented food. Probiotics. Antimicrob. 10, 258–268. 10.1007/s12602-017-9282-x 28527125

[B33] Wang M. Zhu X. L. Wang K. Lu C. G. Luo M. Y. Shan T. L.. (2018). Wheat caffeic acid 3-O-methyltransferase Tacomt-3d positively contributes to both resistance to sharp eyespot disease and stem mechanical strength. Sci. Rep. 8, 6543. 10.1038/s41598-018-24884-0 29695751 PMC5916939

[B34] Wu Z. S. Huang Y. Y. Li Y. Dong J. W. Liu X. C. Li C. (2019). Biocontrol of *Rhizoctonia solani via* induction of the defense mechanism and antimicrobial compounds produced by *Bacillus subtilis* SL-44 on Pepper (*Capsicum annuum* L.). Front. Microbiol. 10, 2676. 10.3389/fmicb.2019.02676 31849858 PMC6892779

[B35] Xu Y. L. Li X. Y. Cong C. Gong G. L. Xu Y. P. Che J.. (2020). Use of resistant *Rhizoctonia cerealis* strains to control wheat sharp eyespot using organically developed pig manure fertilizer. Sci. Total. Environ. 726, 138568. 10.1016/j.scitotenv.2020.138568 32305767

[B36] Yi Y. Luan P. Liu S. Shan Y. Hou Z. Zhao S. Jia S. Li R. (2022). Efficacy of *Bacillus subtilis* XZ18-3 as a biocontrol agent against *Rhizoctonia cerealis* on wheat. Agriculture 12, 258. 10.3390/agriculture12020258

[B37] Zhang Z. X. Wang H. Y. Wang K. Y. Jiang L. L. Wang D. (2017). Use of lentinan to control sharp eyespot of wheat, and the mechanism involved. J. Agr. Food. Chem. 65, 10891–10898. 10.1021/acs.jafc.7b04665 29191011

[B38] Zhao X. L. Song P. Hou D. Y. Li Z. L. Hu Z. J. (2021). Antifungal activity, identification and iosynthetic potential analysis of fungi against *Rhizoctonia cerealis*. Ann. Microbiol. 71, 41. 10.1186/s13213-021-01654-4

[B39] Zhu H. M. Zhao L. N. Zhang X. Y. Foku J. M. Li J. Hu W. C.. (2019). Efficacy of Yarrowia lipolytica in the biocontrol of green mold and blue mold in *Citrus reticulata* and the mechanisms involved. Biol. Control. 139, 104096. 10.1016/j.biocontrol.2019.104096

