# Supplementary material for: Burkholderia ambifaria XN08: A plant growth-promoting endophytic bacterium with biocontrol potential against sharp eyespot in wheat
Source: Front Microbiol. 2022 Jul 28;13:906724. doi: 10.3389/fmicb.2022.906724 (PMC9368319; doi:10.3389/fmicb.2022.906724)

The primers used in this study

| Gene | Primer | Sequences, 5′→3′ PCR product |
| --- | --- | --- |
| *Cep* | *Cep* 1F  *Cep* 1R  Cep 2F  Cep 2R  Cep 3F  Cep 3R | TGGAGCGCTACCAGGAAATG  AGAAAACGGCTCATCAGCGA  AGCTCTTCCAACAGATCGCC  GCACGAGAAAACGGCTCATC  GGAGCGCTACCAGGAAATGA  TCAGATGGTTGATCTCGGCG |
| *Prn* | *Prn* 1F  *Prn* 1R  *Prn* 2F  *Prn* 2R  *Prn* 3F  *Prn* 3R | TCTACCATCTGTTCGGCAGC  AATAGTGCGCCTGGACGAAA  ACCTGTTCATCGACTGCTCC  AAATAGTGCGCCTGGACGAA  TGGTGGCCGATTTCCTGAAG  CAGAAAGCACGACGACAACC |
| *Pca* | *Pca* 1F  *Pca* 1R  *Pca* 2F  *Pca* 2R  *Pca* 3F  *Pca* 3R | *CTCGCGTTCGAGCAGTTGTA*  CATCGTTTTCGAGCGTCAGC  AAAGCCTGTCGATCACGCAG  GCATCGTTTTCGAGCGTCAG  CTCGAAGACGATCGCCGAA ACAGCACCTCGATGAACGAA |

The electrophoregram of PCR products


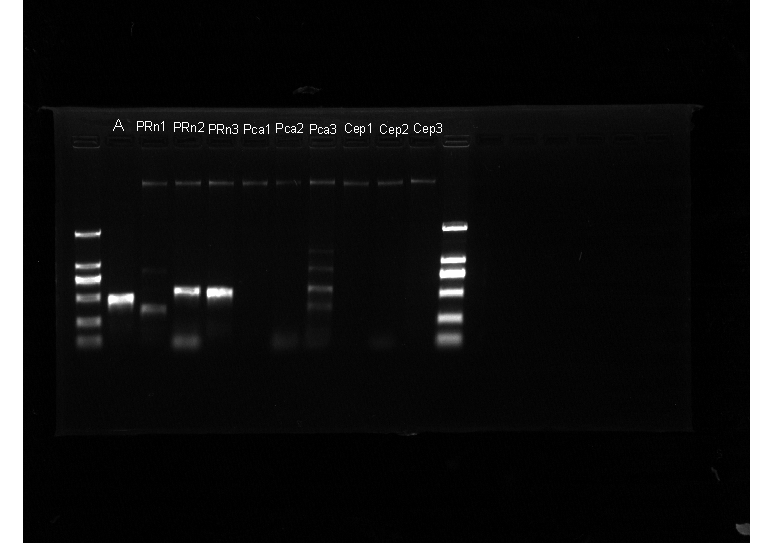


MS spectra of pyrrolnitrin; a same retention times compound corresponded with the standard pyrrolnitrin from strain XN08 (a); standard pyrrolnitrin (b); the *arrow* indicates the molecular ion of pyrrolnitrin at *m*/*z* 256.98.


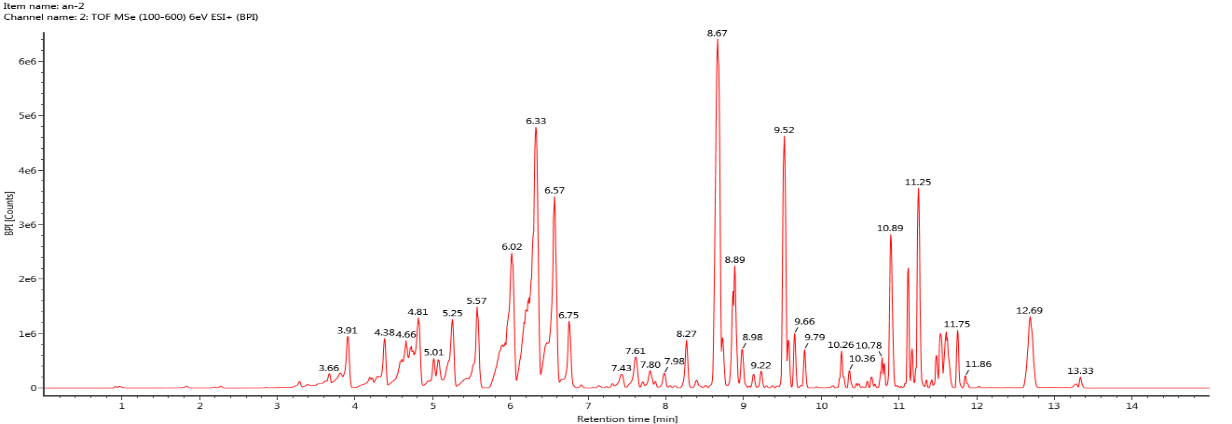

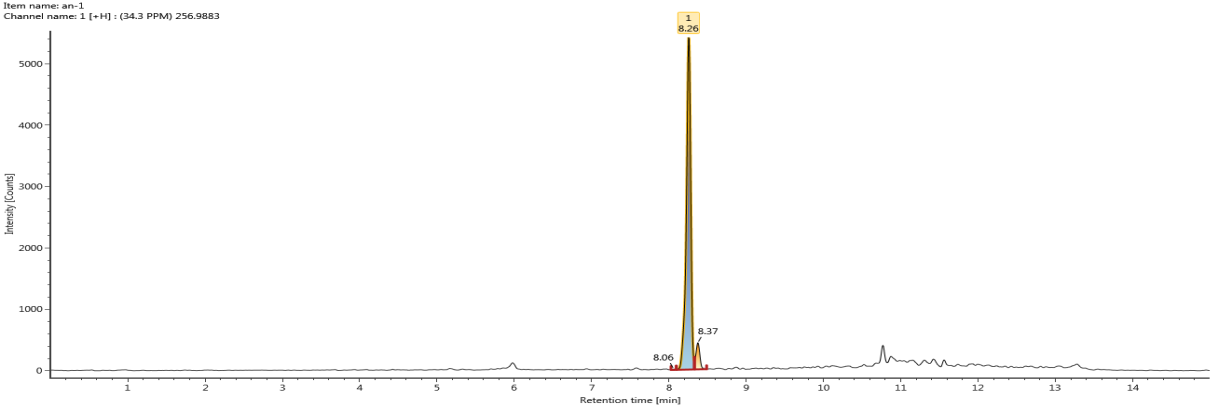


(a)

(b)


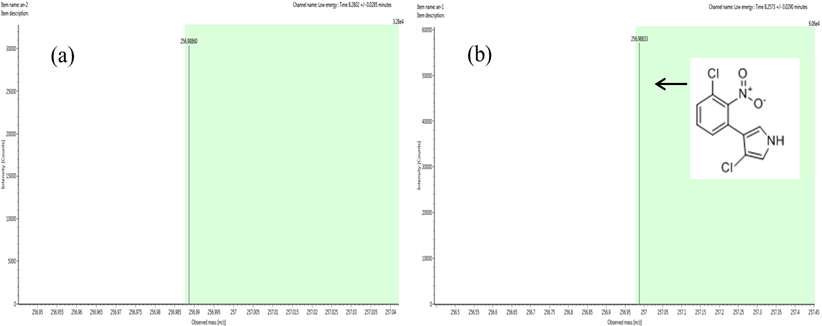

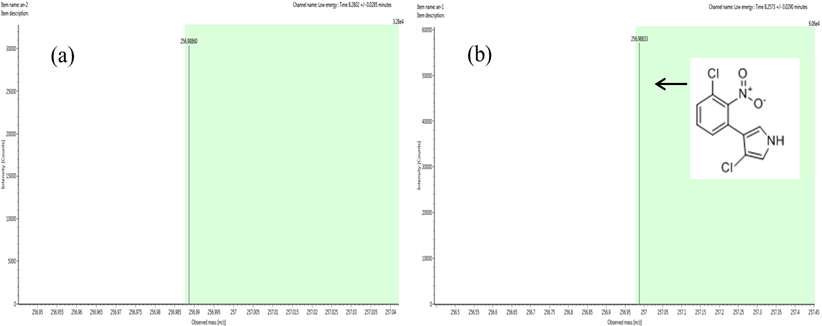

Supplement: Supplementary file 1 [file Data_Sheet_1.docx]
